# Safety and efficacy comparison of single-stage and two-stage ERCP combined with laparoscopic cholecystectomy: a meta-analysis and systematic review

**DOI:** 10.3389/fmed.2025.1713857

**Published:** 2025-11-27

**Authors:** Jian-Xiong Tai, Qingxiang Zhang

**Affiliations:** 1 Hepatobiliary Pancreatic Surgery, Hengshui People’s Hospital in Hebei Province (Harrison International Peace Hospital), Hengshui, China; 2 Department of Hepatobiliary and Pancreatic Surgery, Hengshui People’s Hospital, Hengshui, China

**Keywords:** bile duct stones, ERCP, laparoscopic-endoscopic rendezvous, laparoscopic cholecystectomy, meta-analysis

## Abstract

**Objective:**

This meta-analysis aims to assess the safety and efficacy of single-stage and two-stage ERCP combined with laparoscopic cholecystectomy in the management of bile duct stones.

**Methods:**

A comprehensive search was conducted in PubMed, Embase, Cochrane Library, and Web of Science databases to identify prospective randomized controlled studies comparing the effectiveness of single-stage ERCP combined with laparoscopy, also known as Laparoscopic-Endoscopic Rendezvous (LERV), and the sequential two-step approach of ERCP followed by laparoscopic cholecystectomy (ERCP+LC). Stone clearance success rate, incidence of complications, occurrence of pancreatitis, and hyperamylasemia were analyzed using Stata software.

**Results:**

Nine studies involving a total of 1,003 participants were included in the analysis, with 505 patients undergoing sequential surgery and 498 patients receiving LERV treatment. The LERV group exhibited a significantly higher stone clearance rate compared to the sequential surgery group (RR = 0.62, 95% CI: 0.49–0.79). The incidence of pancreatitis was significantly higher in the sequential surgery group compared to LERV (RR = 1.90, 95% CI: 1.61–2.24). Similarly, the occurrence of hyperamylasemia was significantly higher in the sequential surgery group compared to LERV (RR = 1.93, 95% CI: 1.55–2.40).

**Conclusion:**

The findings of this meta-analysis support the effectiveness of LERV as a treatment option for bile duct stones. LERV demonstrates superior outcomes compared to the sequential two-step approach of ERCP followed by laparoscopic cholecystectomy (ERCP+LC). Specifically, LERV shows improved stone clearance success rates and a decreased incidence of pancreatitis. These results suggest that LERV is a safe and efficient procedure for the management of bile duct stones.

**Systematic review registration:**

2025100075, https://doi.org/10.37766/inplasy2025.10.0075.

## 1 Introduction

Gallstones are the most common disease affecting the biliary system, characterized by the formation of hardened deposits of cholesterol or bilirubin in the bile ducts, varying in size from small particles to large stones (1). Gallstones are commonly associated with biliary infections, particularly parasitic infections, and can cause symptoms such as abdominal pain, nausea, and vomiting, which worsen when bile flow is obstructed (2). Bile stasis and disturbances in cholesterol metabolism are the main contributing factors to stone formation, influenced by various factors (3). Approximately 70% of patients with gallstones are asymptomatic and typically remain so. However, the estimated cumulative incidence rate of symptoms within 10 years in asymptomatic gallstone patients is around 10–20% (4). Studies have shown that gallstones are a risk factor for gallbladder cancer, with relative risks ranging from 2.3 to 34.4 (5). A meta-analysis incorporating seven cohort studies and 23 case–control studies demonstrated that gallstones were considered a major risk factor, associated with stone size and quantity (6).

Cholecystectomy is the preferred treatment for symptomatic gallstones, particularly in patients with recurrent episodes of cholecystitis (7). Laparoscopic cholecystectomy (LC) has largely replaced traditional open surgery as the standard approach (8). Endoscopic retrograde cholangiopancreatography (ERCP) is a minimally invasive technique used for the diagnosis and intervention of biliary and pancreatic diseases. It involves the insertion of an endoscope into the bile and pancreatic ducts, followed by contrast agent injection for imaging purposes (9). ERCP is commonly employed for the diagnosis of biliary and pancreatic diseases, and subsequent interventional treatments based on the diagnostic findings. With the continuous advancement of endoscopic equipment and techniques, the combination of ERCP and LC for the treatment of gallstones has gained wide recognition (10). Sequential two-step procedures involving preoperative ERCP followed by LC (ERCP+LC) remain the mainstay for gallstone management and are recommended by the European Liver Study Association. However, these procedures carry risks of cannulation failure and pancreatitis (11). Single-stage ERCP combined with laparoscopy, also known as laparoscopy-endoscopy rendezvous (LERV), is a novel and appealing technique that has garnered attention for its safety and feasibility (12).

Based on these considerations, this study aims to conduct a meta-analysis evaluating the safety and efficacy of preoperative ERCP combined with LC (sequential surgery) and single-stage LERV.

## 2 Data and methods

### 2.1 Literature search

This study adhered to the rigorous guidelines outlined by the Preferred Reporting Items for Systematic Reviews and Meta-Analyses (PRISMA) for conducting systematic reviews and meta-analyses. A comprehensive literature search was conducted by two independent researchers in the PubMed, Embase, Cochrane Library, and Web of Science databases. The search covered the period from database inception to March 1, 2024. The search strategy employed the following keywords: “laparoscopic cholecystectomy,” “celioscopic cholecystectomy,” “Endoscopic sphincterotomy,” “ERCP,” “endoscopic retrograde cholangiopancreatography,” “EST,” “laparoendoscopic rendezvous,” “LERV,” and “clinical trial.” The search was limited to articles published in English. The exact search strings for each database are provided in the Supplementary material to ensure transparency and reproducibility. Ethical approval was not required for this study, as all data were obtained from previously published articles that had already obtained ethical approval. This meta-analysis was conducted and reported based on Preferred Reporting Items for Systematic Reviews and Meta-Analyses (PRISMA) 2020 checklist. We did not prospectively register this trial, but we have now registered it retrospectively at INPLASY (INPLASY.COM): registration number: 2025100075 DOI number is 10.37766/inplasy2025.10.0075.

### 2.2 Inclusion criteria

This meta-analysis followed the PICOS principle to determine the inclusion of literature. P (Population): patients with gallstones; I (Intervention): laparoscopy-endoscopy rendezvous (LERV) technique; C (Comparison): preoperative ERCP combined with LC (sequential surgery); O (Outcome): success rate of stone clearance, incidence of complications, adverse event rate, length of hospital stay, etc.; S (Study design): prospective randomized controlled trials. Studies that did not meet the intervention criteria, had unclear surgical procedures, incomplete data, or had a sample size of less than 10 were excluded. In studies with the same clinical registration number, the most recent publication was selected.

### 2.3 Data extraction

Two authors independently extracted data and assessed the eligibility of the studies. The extracted information from the original articles included details such as the first author, publication year, country, intervention methods, sample size, patient characteristics, study design, and outcomes. Data extraction was performed using a pre-designed Excel table, and any discrepancies were resolved with the assistance of a third researcher.

### 2.4 Assessment of study quality

The quality of the included studies was evaluated using the risk-of-bias assessment tool developed by the Cochrane Collaboration. This tool examines various criteria, including random sequence generation, allocation concealment, blinding of participants and personnel, blinding of outcome assessment, completeness of outcome data, selective reporting of results, and other potential sources of bias.

Each study was evaluated across seven domains according to the Cochrane Collaboration’s Risk of Bias tool: (1) random sequence generation, (2) allocation concealment, (3) blinding of participants and personnel, (4) blinding of outcome assessment, (5) completeness of outcome data, (6) selective reporting, and (7) other biases.

Overall, most studies demonstrated a low to moderate risk of bias in randomization and outcome completeness. However, blinding of participants and personnel was often unclear or high due to the nature of surgical interventions, and selective reporting could not be fully excluded in several studies.

### 2.5 Statistical analysis

The data analyzed in this study comprised both categorical and continuous outcome variables. Categorical data were analyzed using the risk ratio (RR) along with its corresponding 95% confidence interval (CI). Continuous variables were analyzed using the mean difference (MD) and its 95% CI.

To assess heterogeneity among the studies, the Cochran *Q* test was employed. Heterogeneity was classified as low, moderate, or significant based on the *I*^2^ values: ≤25% for low heterogeneity, 25% ≤ *I*^2^ ≤ 50% for moderate heterogeneity, and *I*^2^ ≥ 50% for significant heterogeneity. For studies with low heterogeneity, a fixed-effects model was used to calculate the pooled effect size. In the case of moderate or significant heterogeneity, a random-effects model was applied.

Publication bias was assessed using a funnel plot, which provides a visual representation of potential bias. Statistical analysis and figure plotting were performed using Stata software. A two-sided *p*-value less than 0.05 was considered statistically significant.

## 3 Results

### 3.1 Literature search process

**Figure 1:**
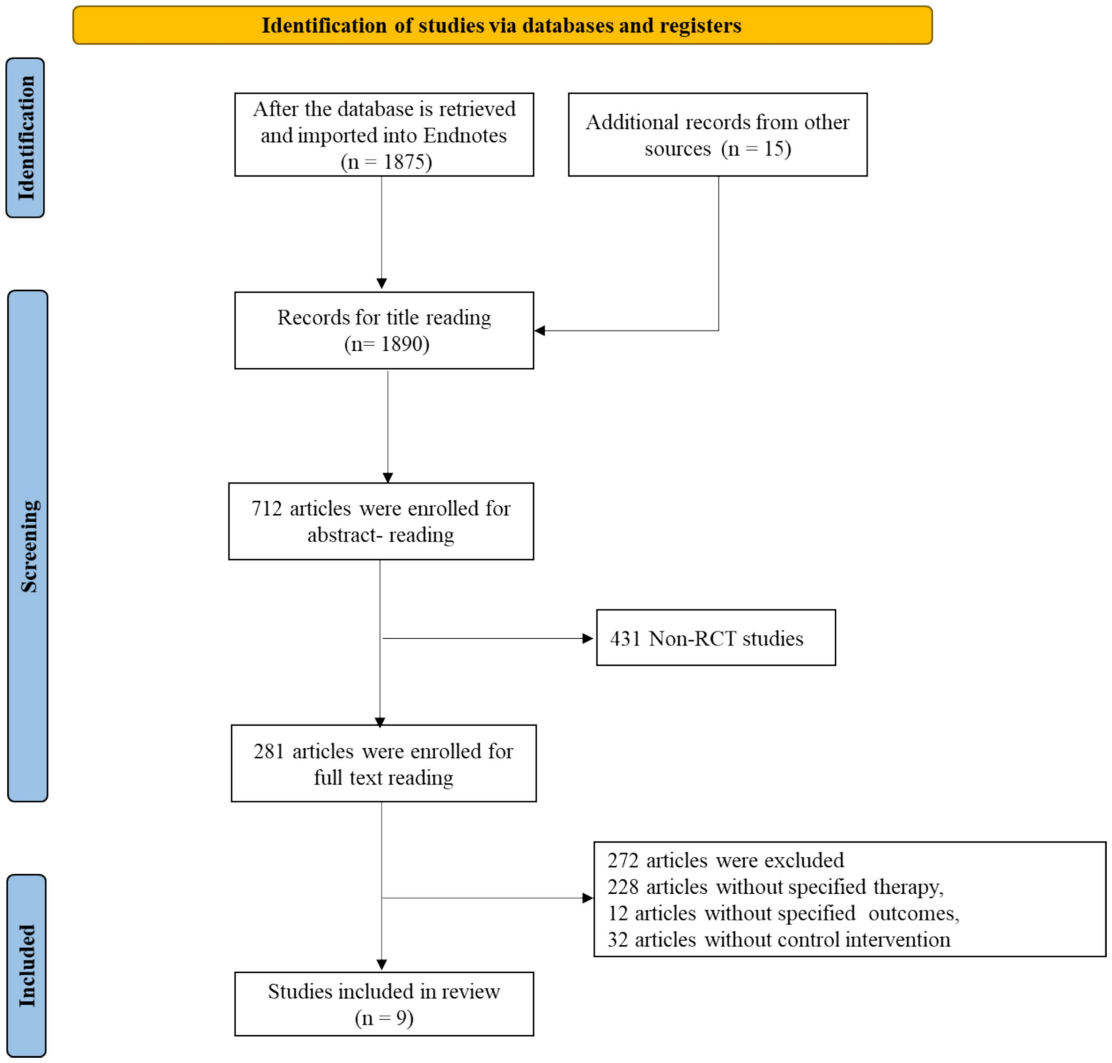
Flowchart of literature selection process.

After conducting a database search and importing the articles into EndNote, a total of 1,890 articles were obtained. After reviewing the abstracts, 1,178 articles that were not clinical studies (such as reviews, case reports, conference papers, and comments) were excluded. The remaining 712 articles underwent full-text reading, resulting in the exclusion of 431 articles that were not prospective randomized controlled trials, 228 articles that were not relevant to the topic, 12 articles from which data could not be extracted, and 32 articles that lacked control groups. Finally, 9 eligible articles were included. The literature search process is shown in Figure 1.

### 3.2 Characteristics of included studies

Among the 9 included studies, a total of 1,003 subjects were enrolled, with 505 patients undergoing sequential surgery and 498 patients undergoing LERV treatment. The outcome measures included the success rate of stone clearance, incidence of complications, occurrence of pancreatitis, and occurrence of hyperamylasemia. The basic information of the included studies is presented in Table 1.

**Table 1:** Basic information of included studies.

Authors	Time	Treatment modalities (control group vs. intervention group)	Cases of control group	Cases of intervention group	Outcome indices
Morino et al. (25)	2006	ERCP+LC vs. LERV	45	46	a, b, c, d
Rábago et al. (26)	2006	ERCP+LC vs. LERV	64	59	a, b, c
Sahoo et al. (27)	2014	ERCP+LC vs. LERV	41	42	a, b, c, d
Tzovaras et al. (28)	2012	ERCP+LC vs. LERV	49	50	a, b, c, d
Lella et al. (29)	2006	ERCP+LC vs. LERV	60	60	b, d
ElGeidie et al. (30)	2011	ERCP+LC vs. LERV	100	98	a, c
Bansal et al. (31)	2010	ERCP+LC vs. LERV	15	15	a, c
Bansal et al. (32)	2014	ERCP+LC vs. LERV	84	84	a, b, c
Pesce et al. (13)	2017	ERCP+LC vs. LERV	47	44	a, c

a, Stone clearance success rate; b, Incidence rate of complications; c, Incidence rate of pancreatitis; d, Incidence rate of hyperamylasemia.

The included studies were published between 2006 and 2017 and were conducted in Europe, Asia, and North Africa. Sample sizes ranged from 30 to 198 patients per study. All trials compared single-stage LERV with two-stage ERCP followed by LC, focusing on outcomes such as stone clearance rate, postoperative complications, pancreatitis, and hyperamylasemia. Most studies adopted a prospective randomized controlled design, with comparable baseline characteristics between groups. Across the studies, the LERV group consistently achieved higher stone clearance success and lower rates of pancreatitis, while overall complication rates were similar. This consistency in study design and outcome measures provides a robust foundation for the subsequent pooled analysis.

**Figure 2:**
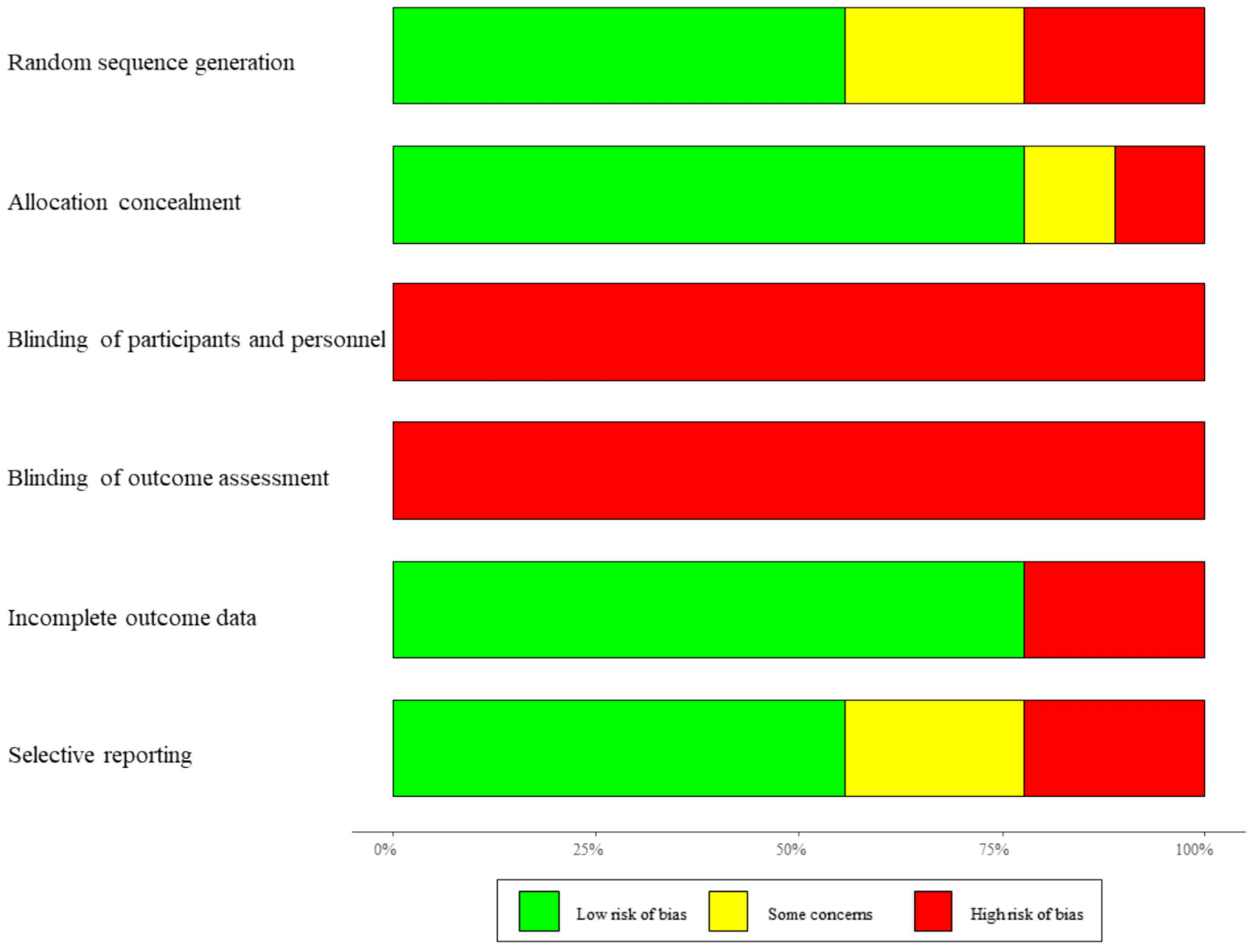
Funnel plot of publication bias.

The quality of the literature was assessed using the Cochrane risk-of-bias assessment tool, and the results are shown in Figure 2. Due to patient informed consent and data collection, there was a high risk of bias in all studies, and the risk of bias in measurement was uncertain.

To further enhance transparency, a detailed per-study risk-of-bias summary is provided in Table 2, outlining the level of risk (low/high/unclear) for each domain in every included study.

**Table 2:** Risk of bias assessment of included studies (Cochrane tool).

Study (Author, Year)	Random sequence generation	Allocation concealment	Blinding of participants & personnel	Blinding of outcome assessment	Incomplete outcome data	Selective reporting	Other bias	Overall risk
Morino et al. (2006) (25)	Low	Unclear	High	Low	Low	Low	Low	Moderate
Rábago et al. (2006) (26)	Low	Low	High	Unclear	Low	Low	Low	Moderate
Sahoo et al. (2014) (27)	Low	Unclear	High	Unclear	Low	Low	Low	Moderate
Tzovaras et al. (2012) (28)	Low	Low	High	Unclear	Low	Low	Low	Moderate
Lella et al. (2006) (29)	Unclear	Unclear	High	Unclear	Low	Low	Low	Moderate
ElGeidie et al. (2011) (30)	Low	Low	High	Low	Low	Low	Low	Low
Bansal VK, 2010 (31)	Low	Low	High	Unclear	Low	Low	Low	Moderate
Bansal et al. (2014) (32)	Low	Low	High	Low	Low	Low	Low	Low
Pesce et al. (2017) (13)	Low	Unclear	High	Unclear	Low	Low	Low	Moderate

### 3.3 Success rate of stone clearance

**Figure 3:**
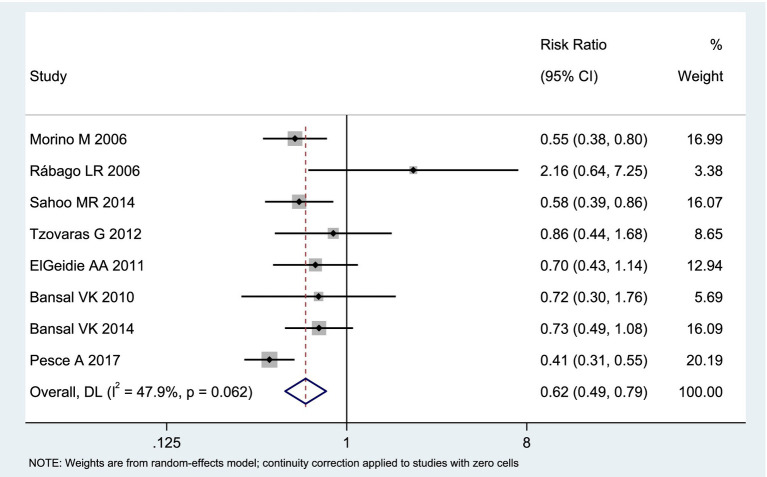
Forest plot of the stone clearance success rate.

**Figure 4:**
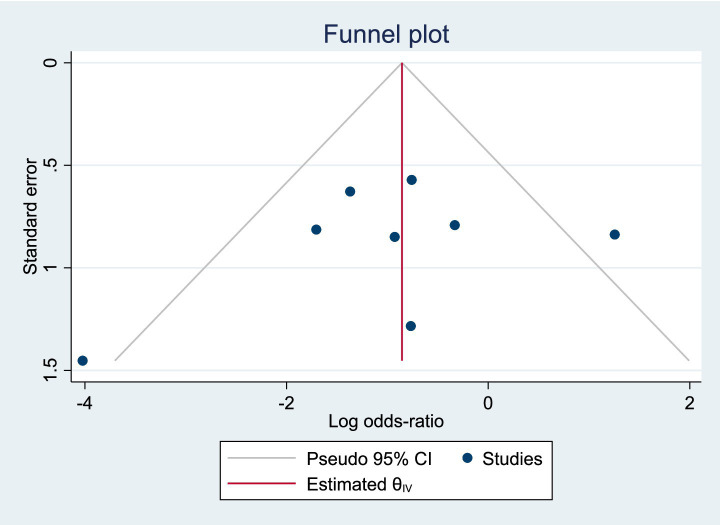
Funnel plot of publication bias for the stone clearance success rate.

Among the included studies, 8 reported the postoperative success rate of stone clearance as an outcome measure, as shown in Figure 3. The analysis of heterogeneity showed moderate heterogeneity (*I*^2^ = 47.9%), and a random-effects model was used for the analysis. Among the 438 patients in the LERV group, stone clearance was successful in 415 patients, while among the 445 patients in the sequential surgery group, stone clearance was successful in 383 patients. The stone clearance rate was significantly higher in the LERV group compared to sequential surgery (RR = 0.62, 95% CI: 0.49–0.79). A funnel plot was used to assess publication bias, and the results shown in Figure 4 indicated a generally symmetrical funnel plot, suggesting no publication bias.

### 3.4 Incidence of complications

**Figure 5:**
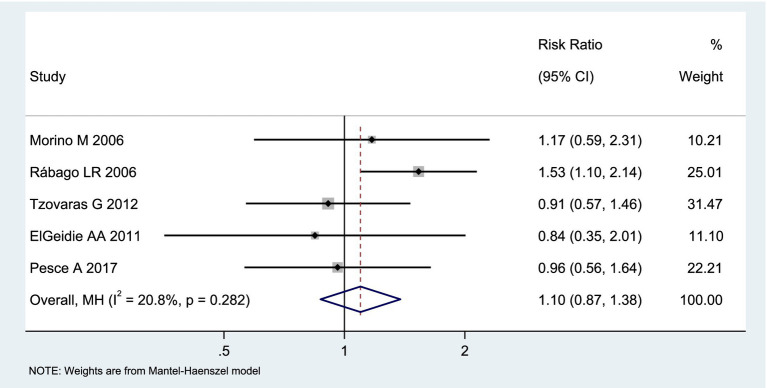
Forest plot of the incidence of complications.

**Figure 6:**
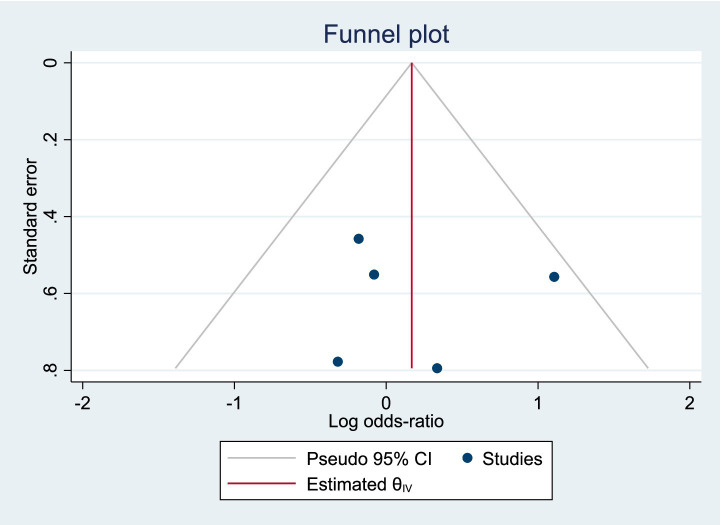
Funnel plot of publication bias for the incidence of complications.

Among the included studies, 5 reported the incidence of postoperative complications as an outcome measure, as shown in Figure 5. The analysis of heterogeneity showed low heterogeneity (*I*^2^ = 20.8%), and a fixed-effects model was used for the analysis. Among the 297 patients in the LERV group, 34 experienced complications, while among the 305 patients in the sequential surgery group, 41 experienced complications. There was no significant difference in the incidence of complications between the two treatment modalities (RR = 1.10, 95% CI: 0.87–1.38). A funnel plot was used to assess publication bias, and the results shown in Figure 6 indicated a generally symmetrical funnel plot, suggesting no publication bias.

### 3.5 Incidence of hyperamylasemia

**Figure 7:**
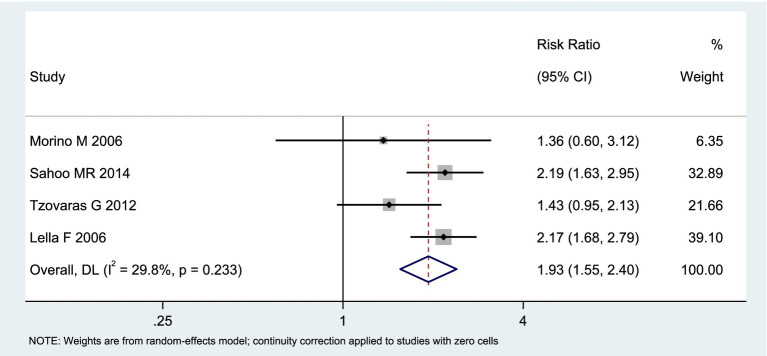
Forest plot of the incidence of hyperamylasemia.

**Figure 8:**
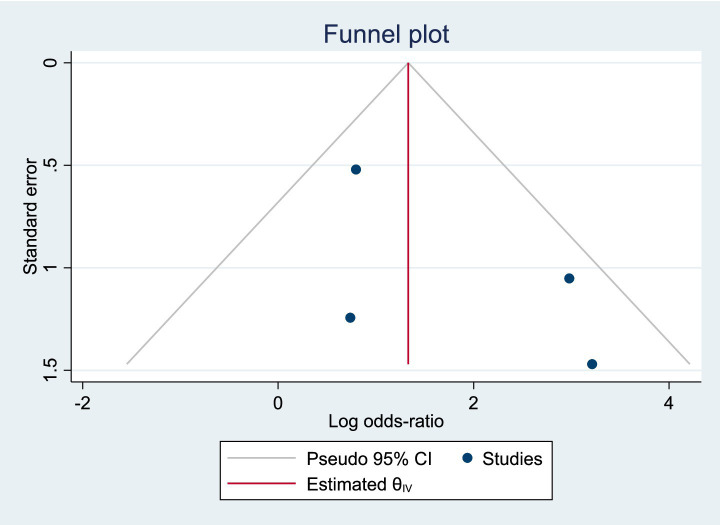
Funnel plot of publication bias for the incidence of hyperamylasemia.

Among the included studies, 4 reported the incidence of hyperamylasemia as an outcome measure, as shown in Figure 7. The analysis of heterogeneity showed moderate heterogeneity (*I*^2^ = 29.8%), and a random-effects model was used for the analysis. Among the 198 patients in the LERV group, 9 experienced hyperamylasemia, while among the 195 patients in the sequential surgery group, 39 experienced hyperamylasemia. The incidence of hyperamylasemia was significantly higher in the sequential surgery group compared to LERV (RR = 1.93, 95% CI: 1.55–2.40). A funnel plot was used to assess publication bias, and the results shown in Figure 8 indicated a generally symmetrical funnel plot, suggesting no publication bias.

### 3.6 Incidence of pancreatitis

**Figure 9:**
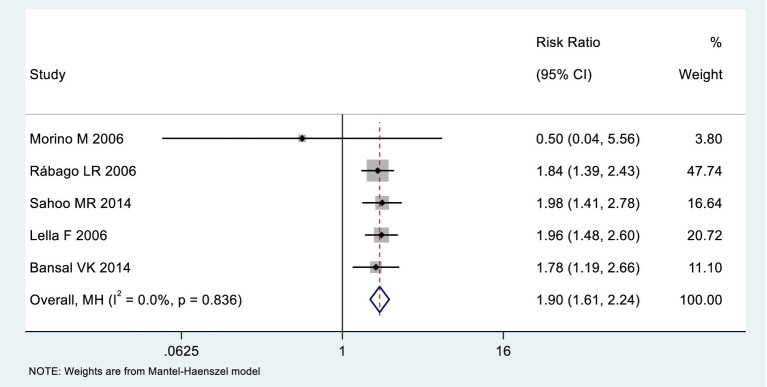
Forest plot of the incidence of pancreatitis hyperplasia.

**Figure 10:**
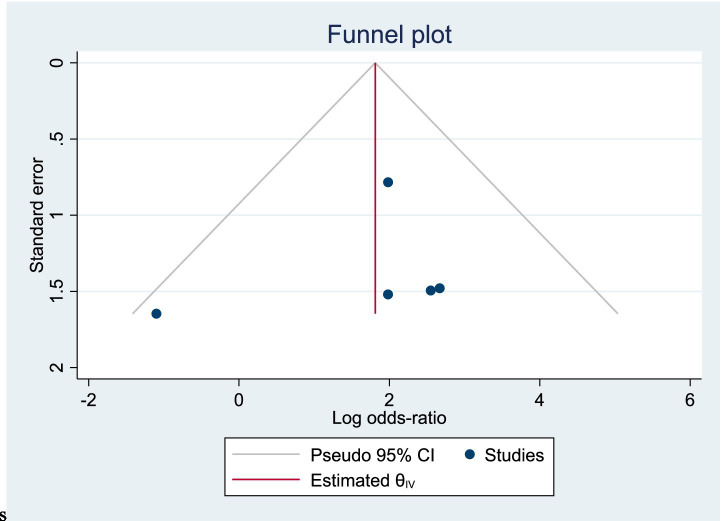
Funnel plot of publication bias for the incidence of pancreatitis.

Among the included studies, 5 reported the incidence of pancreatitis as an outcome measure, as shown in Figure 9. The analysis of heterogeneity showed no heterogeneity (*I*^2^ = 0), and a fixed-effects model was used for the analysis. Among the 291 patients in the LERV group, 3 experienced pancreatitis, while among the 294 patients in the sequential surgery group, 27 experienced pancreatitis. The incidence of pancreatitis was significantly higher in the sequential surgery group compared to LERV (RR = 1.90, 95% CI: 1.61–2.24). A funnel plot was used to assess publication bias, and the results shown in Figure 10 indicated a significantly asymmetrical funnel plot, suggesting significant publication bias.

## 4 Discussion

In this study, among the 438 patients in the LERV group, stone clearance was successful in 415 cases, while in the sequential surgery group of 445 patients, 383 patients achieved successful stone clearance. The stone clearance rate in the LERV group was significantly higher than that in the sequential surgery group (RR = 0.62, 95% CI: 0.49–0.79). In the LERV group, 3 out of 291 patients experienced pancreatitis, whereas in the sequential surgery group of 294 patients, 27 patients developed pancreatitis. The incidence of pancreatitis after sequential surgery was significantly higher than that after LERV (RR = 1.90, 95% CI: 1.61–2.24). High amylase levels are indicative of pancreatitis (13), and in this study, among the 198 patients in the LERV group, 9 patients had amylase elevation, compared to 39 patients in the sequential surgery group. The occurrence of postoperative hyperamylasemia was significantly higher in the sequential surgery group than in the LERV group (RR = 1.93, 95% CI: 1.55–2.40). Endoscopic retrograde cholangiopancreatography (ERCP) offers advantages such as minimal invasiveness, high efficacy, and shorter hospital stays, achieving stone clearance rates of up to 95%. The combination of ERCP and laparoscopic cholecystectomy (LC) has become the mainstay for treating bile duct stones and is widely used in clinical practice (14). Sequential surgery following ERCP has long been the standard treatment for choledocholithiasis (15). However, with advancements in LC techniques, rendezvous techniques, exemplifying LERV, have demonstrated superior advantages (16). This study included 9 prospective randomized controlled trials comparing ERCP sequential surgery and LERV. The analysis revealed that LERV has significant advantages in improving stone retrieval success rates and reducing the incidence of pancreatitis and hyperamylasemia. The effectiveness of ERCP in clearing bile duct stones is undeniable, but it can lead to numerous complications. Mechanical injuries during ERCP, such as catheter insertion, mechanical lithotripsy, and repeated basket stone retrieval, may result in localized inflammation, including pancreatitis, cholangitis, and cholecystitis (17). Pancreatitis is the most common and dreaded complication of ERCP, closely associated with increased pancreatic duct pressure, injury to the bile duct or pancreatic duct, and infection within the bile duct or pancreatic duct (18). The incidence of post-ERCP pancreatitis in high-risk patients exceeds 15%, emphasizing the importance of reducing this occurrence. However, as an invasive procedure, ERCP is complex, with various techniques and high difficulty, and acute pancreatitis after ERCP should not be underestimated (19). A meta-analysis based on 145 RCT studies demonstrated an overall post-ERCP pancreatitis rate of 10.2%, with a rate of 14.1% in high-risk patients, showing no significant change over time (20). A large-scale retrospective study from 2011 to 2017, encompassing 1.2 million ERCP procedures, reported that 4.5% (55,225 cases) resulted in post-ERCP pancreatitis (PEP). The hospitalization rate increased by 15.3%, from 7,735 in 2011 to 8,920 in 2017, and the overall mortality rate rose from 2.8% in 2011 to 4.4% in 2017. Furthermore, therapeutic ERCP was identified as an independent risk factor for PEP (21). The sequence and timing of ERCP and LC remain focal points of discussion. This study confirms that with technological advancements, concurrent intervention can be utilized for the therapeutic management of gallstones and common bile duct stones. The LERV technique, involving wire insertion through the gallbladder duct into the duodenum, guides the endoscope selectively into the common bile duct, significantly reducing the difficulty of ampullary catheterization, enhancing the success rate of catheterization, and lowering the risk of failure in clearing the common bile duct under endoscopy. This approach avoids the risks associated with wire insertion into the pancreatic duct, as well as the swelling and unnecessary contrast agent injection into the pancreatic duct caused by repeated ampullary manipulations, thereby reducing the incidence of pancreatitis (22). Moreover, the funnel plot for pancreatitis outcomes demonstrated significant asymmetry, suggesting the presence of potential publication bias. This finding indicates that smaller studies with negative or non-significant results might have been underreported or unpublished, which could have led to an overestimation of the beneficial effect of LERV in reducing the incidence of pancreatitis. Although the heterogeneity among included studies was low (*I*^2^ = 0%), this potential publication bias requires cautious interpretation of the pooled results. Future meta-analyses should include a larger number of studies, ideally multicenter randomized controlled trials with registered protocols, to validate these findings and minimize the influence of selective reporting.

When treating gallbladder stones combined with common bile duct stones using a combined ERCP and LC approach, ERCP inevitably affects the biliary system. Whether these effects increase the difficulty of subsequent LC is a question worth exploring (23). Currently, most studies suggest that LC should be performed as early as possible after ERCP. However, early inflammatory responses and surgical stress following ERCP may also affect the progress of LC, potentially increasing perioperative complications. To mitigate the impact of ERCP on LC, some literature suggests that performing ERCP for gallbladder stones combined with common bile duct stones concurrently with LC is safe and effective (24), a viewpoint corroborated in this study. The application of LERV also has considerable limitations, as it requires the collaboration of two distinct teams: surgical and endoscopic. This may pose challenges in smaller or community hospitals.

### 4.1 Limitations and future directions

Several limitations of this meta-analysis should be acknowledged. First, although the included randomized controlled trials were generally of good methodological quality, the number of studies was relatively small (*n* = 9), which may limit the robustness and generalizability of the pooled estimates. Second, publication bias may have influenced the results, as indicated by the funnel plot asymmetry for pancreatitis outcomes. Third, variability in surgeon experience, endoscopic expertise, and institutional protocols could have contributed to differences in clinical outcomes among studies. Additionally, blinding of participants and personnel was not feasible due to the nature of surgical interventions, which may have introduced performance bias. Future large-scale, multicenter randomized trials with standardized operative protocols and transparent reporting are warranted to confirm these findings and strengthen the evidence base.

## 5 Conclusion

The findings of this meta-analysis support the effectiveness of LERV as a treatment option for bile duct stones. LERV demonstrates superior outcomes compared to the sequential two-step approach of ERCP followed by laparoscopic cholecystectomy (ERCP+LC). Specifically, LERV shows improved stone clearance success rates and a decreased incidence of pancreatitis. These results suggest that LERV is a safe and efficient procedure for the management of bile duct stones.

## Data Availability

The original contributions presented in the study are included in the article/Supplementary material, further inquiries can be directed to the corresponding author.

## References

[1] Loos M Strobel O Dietrich M Mehrabi A Ramouz A Al-Saeedi M . Hyperamylasemia and acute pancreatitis after pancreatoduodenectomy: two different entities. Surgery. (2021) 169:369–76. doi: 10.1016/j.surg.2020.07.050, PMID: 32981689

[2] Gutt C Schläfer S Lammert F. The treatment of gallstone disease. Dtsch Arztebl Int. (2020) 117:148–58. doi: 10.3238/arztebl.2020.0148, PMID: 32234195 PMC7132079

[3] Sokal A Sauvanet A Fantin B de Lastours V. Acute cholangitis: diagnosis and management. J Visc Surg. (2019) 156:515–25. doi: 10.1016/j.jviscsurg.2019.05.007, PMID: 31248783

[4] Sun H Warren J Yip J Ji Y Hao S Han W . Factors influencing gallstone formation: a review of the literature. Biomolecules. (2022) 12:550. doi: 10.3390/biom12040550, PMID: 35454138 PMC9026518

[5] Ibrahim M Sarvepalli S Morris-Stiff G Rizk M Bhatt A Walsh RM . Gallstones: watch and wait, or intervene? Cleve Clin J Med. (2018) 85:323–31. doi: 10.3949/ccjm.85a.17035, PMID: 29634468

[6] Peponis T Panda N Eskesen TG Forcione DG Yeh DD Saillant N . Preoperative endoscopic retrograde cholangio-pancreatography (ERCP) is a risk factor for surgical site infections after laparoscopic cholecystectomy. Am J Surg. (2019) 218:140–4. doi: 10.1016/j.amjsurg.2018.09.033, PMID: 30473225

[7] Huang D Joo H Song N Cho S Kim W Shin A. Association between gallstones and the risk of biliary tract cancer: a systematic review and meta-analysis. Epidemiol Health. (2021) 43:e2021011. doi: 10.4178/epih.e2021011, PMID: 33541011 PMC8060519

[8] Colvin HS Kimura T Iso H Ikehara S Sawada N Tsugane S. Risk factors for gallstones and cholecystectomy: a large-scale population-based prospective cohort study in Japan. Dig Dis. (2022) 40:385–93. doi: 10.1159/000517270, PMID: 34023821

[9] Abraham S Rivero HG Erlikh IV Griffith LF Kondamudi VK. Surgical and nonsurgical management of gallstones. Am Fam Physician. (2014) 89:795–802. 24866215

[10] Zhang J Li L Jiang Y Li W Li L. Comparative analysis of laparoscopic choledocholithiasis and ERCP treatment after cholecystectomy. BMC Surg. (2023) 23:304. doi: 10.1186/s12893-023-02207-z, PMID: 37803303 PMC10559435

[11] de Meiros KS Aragão Fernandes AC Fulco Gonçalves G Villarim CVO Costa ESLC de Sousa VMC . Cholecystectomy before, simultaneously, or after ERCP in patients with acute cholecystitis: a protocol for systematic review and/or meta analysis. Medicine (Baltimore). (2022) 101:e30772. doi: 10.1097/MD.0000000000030772 36181122 PMC9524974

[12] Sun W Li J Fang J Duan Q He A Lin C. Comparison of efficacy of ERCP+LC and LC+LCBDE on cholecysto-choledocholithiasis and analysis of risk factors for recurrence of choledocholithiasis. Altern Ther Health Med. (2024) 30,7: 103–107. 37944977

[13] Pesce A Lag G Latteri S Guardabasso V Dim F Dib M . Laparo-endoscopic rendez-vous versus sequential "delayed" approach in patients with choledocholithiasis. Minerva Chir. (2017) 72:98–102. doi: 10.23736/S0026-4733.16.07248-5 27981825

[14] Sanders DJ Bomman S Krishnamoorthi R Kozarek RA. Endoscopic retrograde cholangiopancreatography: current practice and future research. World J Gastrointest Endosc. (2021) 13:260–74. doi: 10.4253/wjge.v13.i8.260, PMID: 34512875 PMC8394185

[15] Jones M Johnson M Samourjian E Schlauch K Ozobia N. ERCP and laparoscopic cholecystectomy in a combined (one-step) procedure: a random comparison to the standard (two-step) procedure. Surg Endosc. (2013) 27:1907–12. doi: 10.1007/s00464-012-2647-z 23239300 PMC4050060

[16] Pavlidis ET Pavlidis TE. Current management of concomitant cholelithiasis and common bile duct stones. World J Gastrointest Surg. (2023) 15:169–76. doi: 10.4240/wjgs.v15.i2.169, PMID: 36896310 PMC9988640

[17] Kundumadam S Fogel EL Gromski MA. Gallstone pancreatitis: general clinical approach and the role of endoscopic retrograde cholangiopancreatography. Korean J Intern Med. (2021) 36:25–31. doi: 10.3904/kjim.2020.537, PMID: 33147903 PMC7820643

[18] Obeidat AE Mahfouz R Monti G Kozai L Darweesh M Mansour MM . Post-endoscopic retrograde cholangiopancreatography pancreatitis: what we already know. Cureus. (2022) 14:e21773. doi: 10.7759/cureus.21773, PMID: 35251843 PMC8890589

[19] Bhatt H. Post-endoscopic retrograde cholangiopancreatography pancreatitis: an updated review of current preventive strategies. Clin Exp Gastroenterol. (2021) 14:27–32. doi: 10.2147/CEG.S276361, PMID: 33564256 PMC7866941

[20] Akshintala VS Kanthasamy K Bhullar FA Sperna Weiland CJ Kamal A Kochar B . Incidence, severity, and mortality of post-ERCP pancreatitis: an updated systematic review and meta-analysis of 145 randomized controlled trials. Gastrointest Endosc. (2023) 98:1–6.e12. doi: 10.1016/j.gie.2023.03.023, PMID: 37004815

[21] Mutneja HR Vohra I Go A Bhurwal A Katiyar V Palomera Tejeda E . Temporal trends and mortality of post-ERCP pancreatitis in the United States: a nationwide analysis. Endoscopy. (2021) 53:357–66. doi: 10.1055/a-1220-2242, PMID: 32668463

[22] La Greca G Barbagallo F Sofia M Latteri S Russello D. Simultaneous laparoendoscopic rendezvous for the treatment of cholecystocholedocholithiasis. Surg Endosc. (2009) 24:769–80. doi: 10.1007/s00464-009-0680-3, PMID: 19730946

[23] Arezzo A Vettoretto N Famiglietti F Moja L Morino M. Laparoendoscopic rendezvous reduces perioperative morbidity and risk of pancreatitis. Surg Endosc. (2013) 27:1055–60. doi: 10.1007/s00464-012-2562-3, PMID: 23052536

[24] Lan WF Li JH Wang QB Zhan XP Yang WL Wang LT . Comparison of laparoscopic common bile duct exploration and endoscopic retrograde cholangiopancreatography combined with laparoscopic cholecystectomy for patients with gallbladder and common bile duct stones a meta-analysis of randomized controlled trials. Eur Rev Med Pharmacol Sci. (2023) 27:4656–69. doi: 10.26355/eurrev_202305_32477, PMID: 37259749

[25] Morino M Baracchi F Miglietta C Furlan N Ragona R Garbarini A. Preoperative endoscopic sphincterotomy versus laparoendoscopic rendezvous in patients with gallbladder and bile duct stones. Ann Surg. (2006) 244:889–93. doi: 10.1097/01.sla.0000246913.74870.fc, (discussion 893-886) 17122614 PMC1856638

[26] Rábago LR Vicente C Soler F Delgado M Moral I Guerra I . Two-stage treatment with preoperative endoscopic retrograde cholangiopancreatography (ERCP) compared with single-stage treatment with intraoperative ERCP for patients with symptomatic cholelithiasis with possible choledocholithiasis. Endoscopy. (2006) 38:779–86. doi: 10.1055/s-2006-944617, PMID: 17001567

[27] Sahoo MR Kumar AT Patnaik A. Randomised study on single stage laparo-endoscopic rendezvous (intra-operative ERCP) procedure versus two stage approach (pre-operative ERCP followed by laparoscopic cholecystectomy) for the management of cholelithiasis with choledocholithiasis. J Minim Access Surg. (2014) 10:139–43. doi: 10.4103/0972-9941.134877, PMID: 25013330 PMC4083546

[28] Tzovaras G Baloyiannis I Zachari E Symeonidis D Zacharoulis D Kapsoritakis A . Laparoendoscopic rendezvous versus preoperative ERCP and laparoscopic cholecystectomy for the management of cholecysto-choledocholithiasis: interim analysis of a controlled randomized trial. Ann Surg. (2012) 255:435–9. doi: 10.1097/SLA.0b013e3182456ec0, PMID: 22261836

[29] Lella F Bagnolo F Rebuffat C Scalambra M Bonassi U Colombo E. Use of the laparoscopic-endoscopic approach, the so-called “rendezvous” technique, in cholecystocholedocholithiasis: a valid method in cases with patient-related risk factors for post-ERCP pancreatitis. Surg Endosc. (2006) 20:419–23. doi: 10.1007/s00464-005-0356-6, PMID: 16424987

[30] ElGeidie AA ElEbidy GK Naeem YM. Preoperative versus intraoperative endoscopic sphincterotomy for management of common bile duct stones. Surg Endosc. (2011) 25:1230–7. doi: 10.1007/s00464-010-1348-8, PMID: 20844893

[31] Bansal VK Misra MC Garg P Prabhu M. A prospective randomized trial comparing two-stage versus single-stage management of patients with gallstone disease and common bile duct stones. Surg Endosc. (2010) 24:1986–9. doi: 10.1007/s00464-010-0891-7, PMID: 20135172

[32] Bansal VK Misra MC Rajan K Kilambi R Kumar S Krishna A . Single-stage laparoscopic common bile duct exploration and cholecystectomy versus two-stage endoscopic stone extraction followed by laparoscopic cholecystectomy for patients with concomitant gallbladder stones and common bile duct stones: a randomized controlled trial. Surg Endosc. (2014) 28:875–85. doi: 10.1007/s00464-013-3237-4 24162138

