# Supplementary material for: Safety and efficacy comparison of single-stage and two-stage ERCP combined with laparoscopic cholecystectomy: a meta-analysis and systematic review
Source: Front Med (Lausanne). 2025 Nov 27;12:1713857. doi: 10.3389/fmed.2025.1713857 (PMC12695829; doi:10.3389/fmed.2025.1713857)
Supplement: Supplementary file 2 [file Table_2.docx]

### ****Search Strategy Transparency****

A detailed search strategy was developed to ensure transparency and reproducibility. The literature search covered **from database inception to March 1, 2024**, and was conducted independently by two reviewers across **PubMed, Embase, Cochrane Library, and Web of Science**. The exact search strings used for each database are listed below.

| **1. PubMed Search Strategy** |
| --- |
| (("laparoscopic cholecystectomy"[MeSH Terms] OR "celioscopic cholecystectomy"[All Fields])  AND ("endoscopic retrograde cholangiopancreatography"[MeSH Terms] OR "ERCP"[All Fields] OR "endoscopic sphincterotomy"[All Fields] OR "EST"[All Fields])  AND ("laparoendoscopic rendezvous"[All Fields] OR "LERV"[All Fields])  AND ("randomized controlled trial"[Publication Type] OR "clinical trial"[Publication Type])) |
| Filters: English; Humans |
| Timeframe: from database inception to March 1, 2024 |
| **2. Embase Search Strategy** |
| ('laparoscopic cholecystectomy'/exp OR 'celioscopic cholecystectomy':ab,ti)  AND ('endoscopic retrograde cholangiopancreatography'/exp OR ercp:ab,ti OR 'endoscopic sphincterotomy':ab,ti OR est:ab,ti)  AND ('laparoendoscopic rendezvous':ab,ti OR lerv:ab,ti)  AND ('randomized controlled trial'/exp OR 'clinical trial'/exp)  AND [english]/lim |
| Timeframe: from database inception to March 1, 2024 |
| **3. Cochrane Library Search Strategy** |
| (laparoscopic cholecystectomy OR celioscopic cholecystectomy)  AND (endoscopic retrograde cholangiopancreatography OR ERCP OR endoscopic sphincterotomy OR EST)  AND (laparoendoscopic rendezvous OR LERV)  In Trials (Word variations have been searched) |
| Timeframe: from database inception to March 1, 2024 |
| **4. Web of Science Search Strategy** |
| TS=("laparoscopic cholecystectomy" OR "celioscopic cholecystectomy")  AND TS=("endoscopic retrograde cholangiopancreatography" OR "ERCP" OR "endoscopic sphincterotomy" OR "EST")  AND TS=("laparoendoscopic rendezvous" OR "LERV")  AND TS=("clinical trial" OR "randomized controlled trial")  Refined by: Language=(English) |
| Timespan: from database inception to March 1, 2024 |

Note: Two independent reviewers performed the literature screening and data extraction process using a pre-piloted data collection form. Any discrepancies were resolved through discussion until a consensus was reached.
